# Dengue Virus Infection Mediates HMGB1 Release from Monocytes Involving PCAF Acetylase Complex and Induces Vascular Leakage in Endothelial Cells

**DOI:** 10.1371/journal.pone.0041932

**Published:** 2012-07-30

**Authors:** Siew Pei Ong, Ling Min Lee, Yew Fai Ivan Leong, Mah Lee Ng, Justin Jang Hann Chu

**Affiliations:** Laboratory of Molecular RNA Virology and Antiviral Strategies, Department of Microbiology, Yong Loo Lin School of Medicine, National University of Singapore, Singapore, Singapore; University of Rochester, United States of America

## Abstract

High mobility group box 1 (HMGB1) protein is released from cells as a pro-inflammatory cytokine in response to an injury or infection. During dengue hemorrhagic fever (DHF)/dengue shock syndrome (DSS), a number of pro-inflammatory cytokines are released, contributing to disease pathogenesis. In this study, the release of HMGB1 from human myelogenous leukemia cell line K562 and primary peripheral blood monocytes (PBM) cells was examined during dengue virus (DV)-infection. HMGB1 was shown to translocate from cell nuclei to the cytoplasm in both K562- and PBM-infected cells. The translocation of HMGB1 from the nucleus to the cytoplasm was shown to be mediated by the host cell p300/CBP-associated factor (PCAF) acetylase complex in K562 cells. In addition, DV capsid protein was observed to be the putative viral protein in actuating HMGB1 migration from the nucleus to cytoplasm through the involvement of PCAF acetylase. HMGB1 was released from DV-infected K562 cells into the extracellular milieu in a multiplicity of infection (M.O.I.)-independent manner and its release can be inhibited by the addition of 1–5 mM of ethyl pyruvate (EP) in a dose-dependent manner. Application of DV-infected K562 cell culture supernatants to primary endothelial cells induced vascular permeability. In contrast, supernatants from DV-infected K562 cells treated with EP or HMGB1 neutralizing antibody were observed to maintain the structural integrity of the vascular barrier.

## Introduction

Dengue virus (DV) is an enveloped, single-stranded, positive-sense RNA virus with a genome of approximately 10.9 Kb. The four distinct serotypes of DV (DV1-4) belong to the genus *Flavivirus* within the family *Flaviviridae* [1]. In humans, DV infection results in a diversity of clinical manifestations ranging from the mild dengue fever (DF) and to the severe dengue hemorrhagic fever (DHF)/dengue shock syndrome (DSS) which is characterized by plasma leakage and thrombocytopenia [2].

While several risk factors associated with DHF/DSS have been identified, the pathogenesis of DHF/DSS is still poorly understood. There is strong evidence implicating the involvement of the immune response in the progression to DHF/DSS, with antibodies, T cells, and cytokines all playing a role [2]. During a secondary DV infection, antibody-dependent enhancement (ADE) can lead to DHF/DSS, whereby pre-existing, heterotypic antibodies bind to infecting virus. Rather than neutralizing viral activity, antibodies bound to virus cause an enhanced infection [3], [4], [5]. This results in a higher viral load in patients, especially in the early stages of infection, and thus an increased risk in developing DHF/DSS [6]. In addition, anti-DV antibodies have been observed to cross-react with platelets and the endothelium, disrupting both blood clotting and vascular systems [7], [8].

During a secondary DV infection, memory T cells derived from a primary infection recognize viral antigens of the current infecting DV serotype with a lower affinity. These memory T cells undergo clonal expansion and dominate the T cell population [9], [10], [11]. The expanded T cell population is unable to clear the infection effectively, and the high viral load results in the induction of a strong pro-inflammatory cytokine response, increasing the risk of DHF/DSS [10], [12], [13].

Analysis of serum from DHF/DSS patients shows elevated levels of IL-8 [14], IL-10 [15], IL-12 [16], TNF-α [17], IFN-α [18], IFN-γ [19], soluble IL-2 receptors and soluble TNF receptors [20]. In addition, cytokines such as IL-2 [21], IL-6 [22], IL-8 [23], TNF-α [24], IL-1α and IL-1β [25] have been shown to mediate vascular permeability.

High mobility group box 1 protein (HMGB1) is a 25 KDa, non-histone, nucleosomal protein [26]. In a normal cell, the protein is predominantly localized in the nucleus where it regulates transcription [27]. HMGB1 protein can be released by cells into the extracellular milieu to function as a proinflammatory cytokine in response to injury, infection and inflammation [28]. Monocytes [29], macrophages, mature dendritic cells (DC) [30] and natural killer (NK) cells [31] are all examples of cell types which actively release HMGB1 protein upon cellular stimulation by signals such as lipopolysaccharide (LPS), proinflammatory stimuli [30] and mycobacterium infection [32]. Acting as a cytokine, HMGB1 protein stimulates monocytes, macrophages and DC to release proinflammatory cytokines TNF-α, Il-6, IL-8, IL-12, MIP-1α, MIP-1β, IL-1α, IL-1β and IL-1RA [33], [34]. In addition, HMGB1 protein also stimulates the endothelial cells to induce the expression of adhesion molecules and release of cytokines [35].

HMGB1 was recently shown to be released from epithelial cells during DV-induced necrosis [36]. In DV-infected DCs, HMGB1 was also released from cells, even under non-necrotic conditions [37]. Similarly, HMGB1 may also be involved in DV-infection of monocytes, contributing to the pathogenesis of DHF/DSS through the disruption of endothelial cell integrity [38]. In this study, the release of HMGB1 from DV-infected monocytes was investigated, using K562 and peripheral blood monocytes (PBM) cells. K562 is a suspension cell line derived from a patient with chronic myeloid leukemia that exhibits characteristic properties of monocytes [39] and the suitability of this cell line for use in this study was previously shown by Chen *et al* (2008) [40]. PBM obtained from healthy blood donors were also included in this study. The usage of PBM allows for the comparison of HMGB1 release to be made to K562 cell line. Our studies revealed that DV induced the migration of HMGB1 from the nucleus to the cytosol and release of HMGB1 into extracellular milieu of both K562 and PBM cells. This process can be inhibited by ethyl pyruvate (EP) or HMGB1 neutralizing antibody. In addition, host cell p300/CBP-associated factor (PCAF) acetylase complex was shown to mediate HMGB1 translocation during DV-infection in K562 cells. HMGB1 released from DV-infected K562 cells was observed to trigger the reduction of vascular integrity in primary HUVEC, which can be prevented with the use of EP. For the first time, we have also identified DV capsid protein as the putative viral protein in mediating HMGB1 release in K562 cells.

## Results

### Dengue Virus Infection Induces the Release of HMGB1 from K562 and PBM Cells

**Figure 1:**
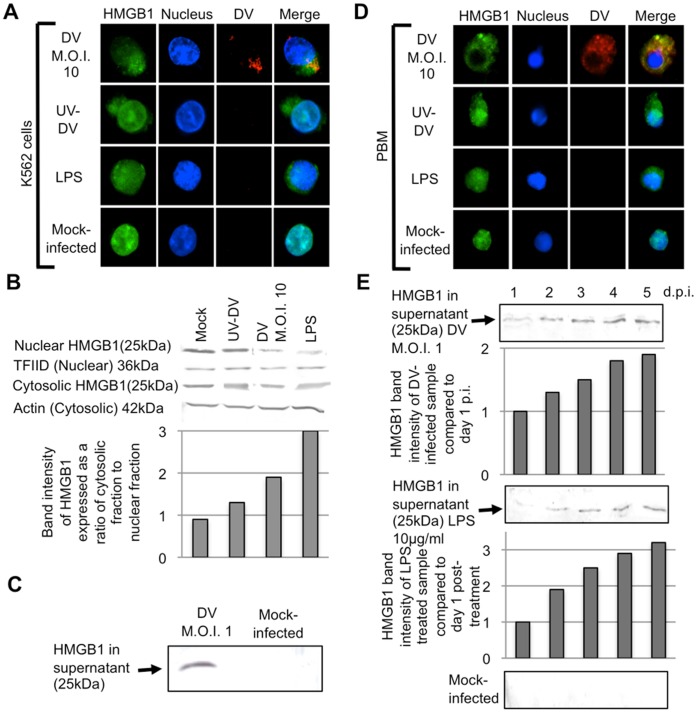
DV induces translocation of HMGB1 from cell nuclei to cytoplasm and into the extracellular milieu. A, Immunofluorescence analyses (IFA) were performed on DV-infected K562. K562 cells were infected at M.O.I. 10 and fixed at 3 d.p.i. HMGB1 was stained green with rabbit anti-HMGB1 antibody and goat anti-rabbit IgG FITC conjugated antibody while DV was stained red using mouse anti-E antibody and anti-mouse-IgG Alexa Fluor 532. The cell nuclei were stained blue with DAPI. B, K562 cells were mock-infected, treated with UV-irradiated DV (UV-DV) at a M.O.I. 10, infected with DV (M.O.I. 10), or treated with 5 µg/ml of LPS. The nuclear and cytosolic fractions were harvested at 3 d.p.i. and Western blot analysis was performed to detect for HMGB1. The band intensities of HMGB1 of the mock-infected, UV-DV treated, DV-infected and LPS-stimulated nuclear fractions were expressed as a ratio to its respective cytosolic fraction. The housekeeping proteins TFIID and actin were included as loading controls for the nuclear and cytosolic fractions, respectively. C, K562 cells were infected with DV at M.O.I. of 1. Cell culture supernatants from DV-infected K562 were harvested at 3 d.p.i. and concentrated for the detection of HMGB1 via Western blot. D, DV-infected PBM cells infected at M.O.I. 1 and the cells fixed at 3 d.p.i. for IFA. HMGB1 was stained green with rabbit anti-HMGB1 antibody and goat anti-rabbit IgG FITC conjugated antibody while DV was stained red using mouse anti-E antibody and anti-mouse-IgG Alexa Fluor 532. The cell nuclei were stained blue with DAPI. E, PBM were infected with DV at a M.O.I. of 1 and Western blot analysis of HMGB1 in the cell culture supernatants of DV-infected PBM was performed. The band intensities of HMGB1 from DV-infected sample and LPS-treated cells at day 1 p.i. were assigned to a value of 1. The relative fold difference in the intensities of DV-infected samples and LPS-treated cells from day 2- to 5 p.i. was measured in relation to the band intensity its respective treatment group at day 1 p.i.

Initial experiments were performed to determine whether DV-infection induces the translocation of HMGB1 from the nucleus to the cytoplasm in K562 cells. The cells were infected at a M.O.I. of 10 to increase the infection rate (Fig. 1a and b) Immunofluorescence analyses (IFA) were performed DV-infected K562 cells to assess the migration of HMGB1 from the nucleus to the cytoplasm of DV-infected cells and representative images are shown in Fig. 1a. HMGB1 was also observed in the cytoplasm of DV-infected K562 cells hence, suggesting that the export of HMGB1 from the nucleus to the cytoplasm upon DV infection. K562 cells incubated with UV-irradiated virus (UV-DV) displayed a similar staining pattern as the cells stimulated with LPS, a positive control (Gardella *et al.* 2002), with the majority of HMGB1 seen in cytoplasmic regions. In contrast, HMGB1 remained in the nucleus of the mock-infected cells.

To corroborate that DV infection actuates the translocation of HMGB1 protein from the nuclei to cytoplasm of the DV-infected cells, Western blot analyses were carried out on nuclear and cytosolic fractions of K562 cells infected with DV for 3 days to detect for the presence of HMGB1. As shown in Fig. 1b, cytosolic fractions of DV-infected cells contain 90% more HMGB1 than nuclear fractions, suggesting that HMGB1 migrates from the nucleus to the cytoplasm upon DV-infection. Similarly, K562 cells incubated with UV-irradiated DV showed an accumulation of HMGB1 in the cytosol. In contrast, there was 10% more HMGB1 in the nuclear fraction of mock-infected cells than in cytosolic fractions, consistent with previous reports that HMGB1 equilibrium is shifted towards nuclear accumulation in normal cells [29]. K562 cells stimulated with LPS showed similar HMGB1 accumulation pattern as the DV-infected cells.

To examine if DV was able to induce the release of HMGB1 from the intracellular cytoplasm to extracellular in milieu at a lower M.O.I. of 1, Western blots were performed on concentrated cell supernatants at 3 d.p.i. As shown in Fig. 1c, HMGB1 was detected in the cell culture supernatants of DV-infected cells and this confirms that DV infection can induce the release of HMGB1 from the nucleus to extracellular milieu. In contrast, HMGB1 was not detected in the supernatant of mock-infected K562 cells at 3 d.p.i.

As K562 cells showed HMGB1 release upon DV-infection, we went on to investigate if DV-infection of PBM cells from healthy blood donors showed similar HMGB1 translocation. PBM cells were infected at M.O.I. of 1 and similar to DV-infected K562 cells, HMGB1was observed in the cytoplasm of DV-infected PBM cells (Fig. 1d). Thus, indicating the export of HMGB1 from the nucleus to the cytoplasm. In addition, PBM cells treated with UV-DV or LPS also showed HMGB1 translocation from the nucleus to the cytoplasmic compartment.

Comparable to K562 cells, HMGB1 was detected in the cell culture media of DV-infected PBM and the amount of protein accumulated over time increased and there was a 90% increment in HMGB1 as the infection progressed from day 1 to 5 (Fig. 1e). LPS-treated PBM showed similar trend as the DV-infected counterparts with increasing amount of HMGB1 detected in the cell culture supernatant. No HMGB1 was detected from the mock-infected monocyte cell culture supernatants. Hence DV-infection in both K562 cells and PBM induces the release of HMGB1 from the nuclei to the extracellular environment.

### Ethyl Pyruvate (EP) Inhibits the Release of HMGB1 from K562 Cells and PBM during DV Infection

**Figure 2:**
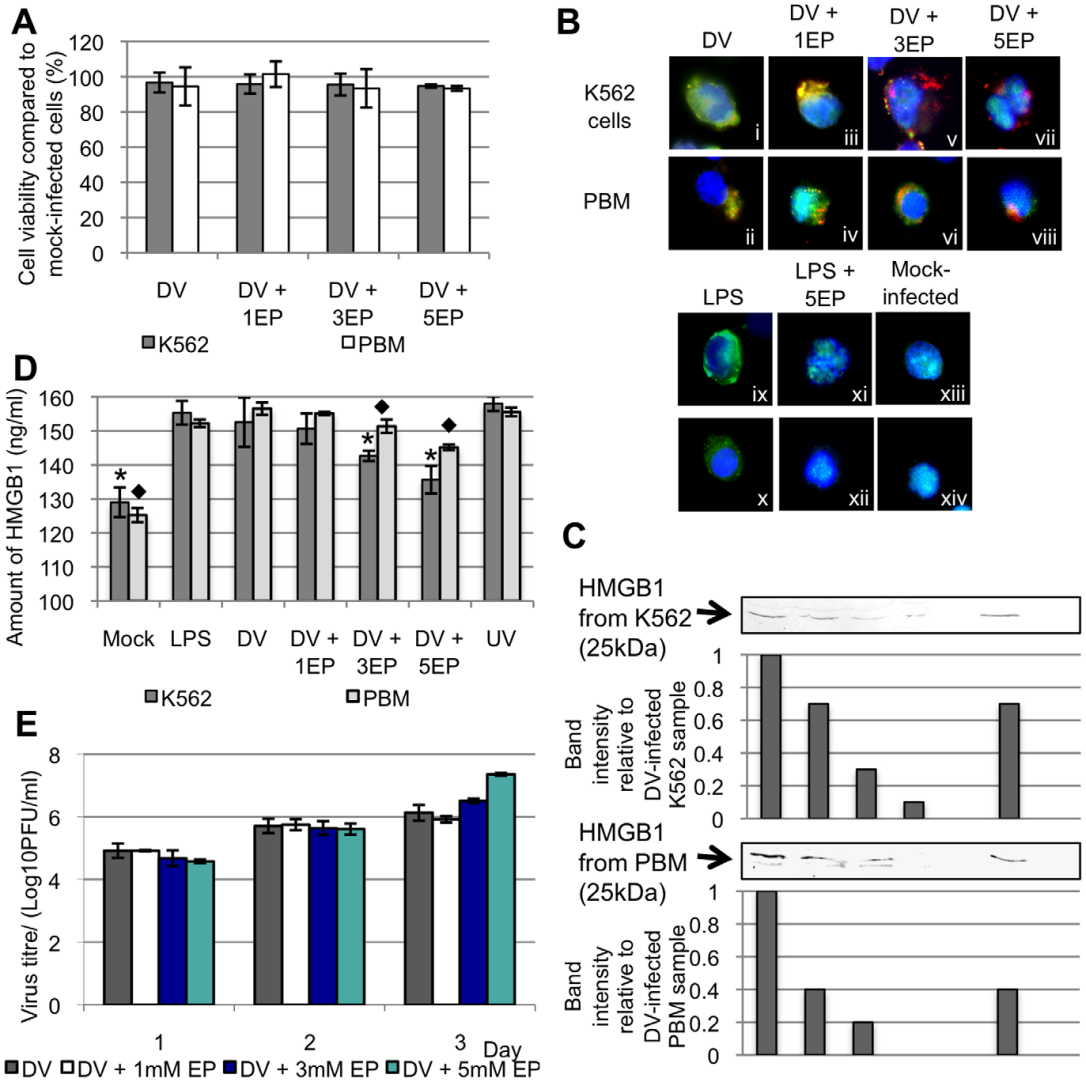
Ethyl pyruvate inhibits HMGB1 release from DV-infected cells in dose-dependent manner. A, cell viability assay of K562 or PBM cells treated with 1, 3 and 5 mM of EP for 3 days. The percentages of viable K562 and PBM cells were calculated in relation to its untreated control groups. B, immunofluorescence analysis of EP-treated DV-infected K562 and PBM cells to visualize the sub-cellular localization of HMGB1 (stained green) in these cells. DV antigens were stained red and the cell nuclei were stained blue. C, K562 and PBM cells were infected with DV (M.O.I. of 1) and the cells were treated with EP at 0 hr p.i. Cell culture supernatants of DV-infected K562 and PBM cells were harvested 3 d.p.i. and concentrated for the detection of HMGB1 by Western blotting. The fold intensity of DV-infected K562 and PBM cells without EP treatment were assigned with a value of 1. The relative fold difference in the HMGB1 intensities of EP-treated DV-infected samples, mock-infected and LPS-treated cells were compared in relation to that of the DV-infected K562 or PBM cells. D, amount of HMGB1 in the cell culture supernantants was quantified using ELISA. * denotes p-value <0.05 for T-tests comparing the mean amount of HMGB1 detected in K562 cell culture supernatants to that of the DV-infected K562 cells. ⧫ denotes p-value <0.05 for T-tests comparing the mean amount of HMGB1 detected in PBM cell culture supernatants to that of the DV-infected PBM cells. E, plaque assays were conducted on the cell culture supernatants of DV-infected EP-treated K562 cells at 3 d.p.i.

Ethyl pyruvate has been shown to inhibit HMGB1 release from LPS-stimulated macrophages [41], [42]. Therefore, EP was investigated for its ability to inhibit HMGB1 release from dengue-infected K562 and PBM cells. Firstly, cell viability assays were first performed to assess the cytotoxicity of EP on K562 or PBM cells. Cell viability assays indicate DV-infection alone resulted in a reduction of less than 6% cell viability for both K562 and PBM cells. Similarly, there is no significant cytotoxicity induced (less than 10%) in K562 and PBM cells at the 3 concentrations of EP tested (Fig. 2a).

Immunofluorescence analyses of EP-treated, DV-infected K562 and PBM cells were performed to visualize the subcellular localization of HMGB1 in these cells (Fig. 2b). DV-infection (Fig. 2bi and ii) or LPS stimulation (Fig. 2bix and x) of K562 and PBM cells resulted in translocation of HMGB1 from nuclei to the cytoplasm. Treatment of DV-infected K562 and PBM cells with increasing concentrations of EP progressively inhibited the translocation of HMGB1 from the nucleus to the cytoplasm (Fig. 2biii to viii). At 5 mM EP, the majority of HMGB1 remained localized in the DV-infected cell nuclei (Fig. 2bvii and viii). Similarly, LPS-stimulated PBM treated with 5 mM EP also showed an inhibition of HMGB1 migration to from cell nuclei to the cytoplasm (Fig. 2bxi and xii).

To corroborate EP could inhibit HMGB1 release into extracellular milieu upon DV infection, DV-infected K562 and PBM cells were incubated with 1 mM, 3 mM or 5 mM of EP. Three d.p.i., cell culture supernatants were harvested. Western blots and ELISA were performed to detect and quantify the amount of HMGB1 in cell culture supernatants. As shown in Fig. 2c, ethyl pyruvate inhibited the release of HMGB1 from both DV-infected K562 and PBM cells in a dose-dependent manner. At 5 mM of EP, the amount of HMGB1 was drastically reduced to ∼10% or less in K562 and PBM cells. In addition, 5 mM of EP inhibited the release of HMGB1 from LPS-stimulated cells, suggesting that there may be similarities in the mechanisms by which EP limits HMGB1 release from LPS-stimulated and DV-infected cells. Similarly, the amount of HMGB1 as detected using ELISA showed a significant reduction in HMGB1 from DV-infected monocytes with 3 and 5 mM of EP used (Fig. 2d).

To exclude the possibility of EP in inhibiting DV replication and affecting HMGB1 release, plaque assays were performed on supernatants collected from EP-treated, DV-infected K562 cells from 1 to 3 d.p.i. It was apparent that EP treatment did not cause a reduction in virus titre compared to untreated samples (Fig. 2d), indicating that EP inhibits HMGB1 release without affecting DV replication.

As DV-infection of both K562 and PBM cells yielded comparable observation on HMGB1 release that could be prevented by EP, we propose that both K562 and PBM cells would probably share similar HMGB1 secretory pathway during DV-infection. Therefore, K562 cells was further used to examine the effect of HMGB1 released from DV-infected monocytes have on vascular permeability as well as to investigate on the molecular pathway involved in mediating HMGB1 release which may reflect the *in vivo* state of monocytes during DV-infection.

### HMGB1 Released from DV-infected K562 Cells Induces Vascular Leakage in HUVEC

**Figure 3:**
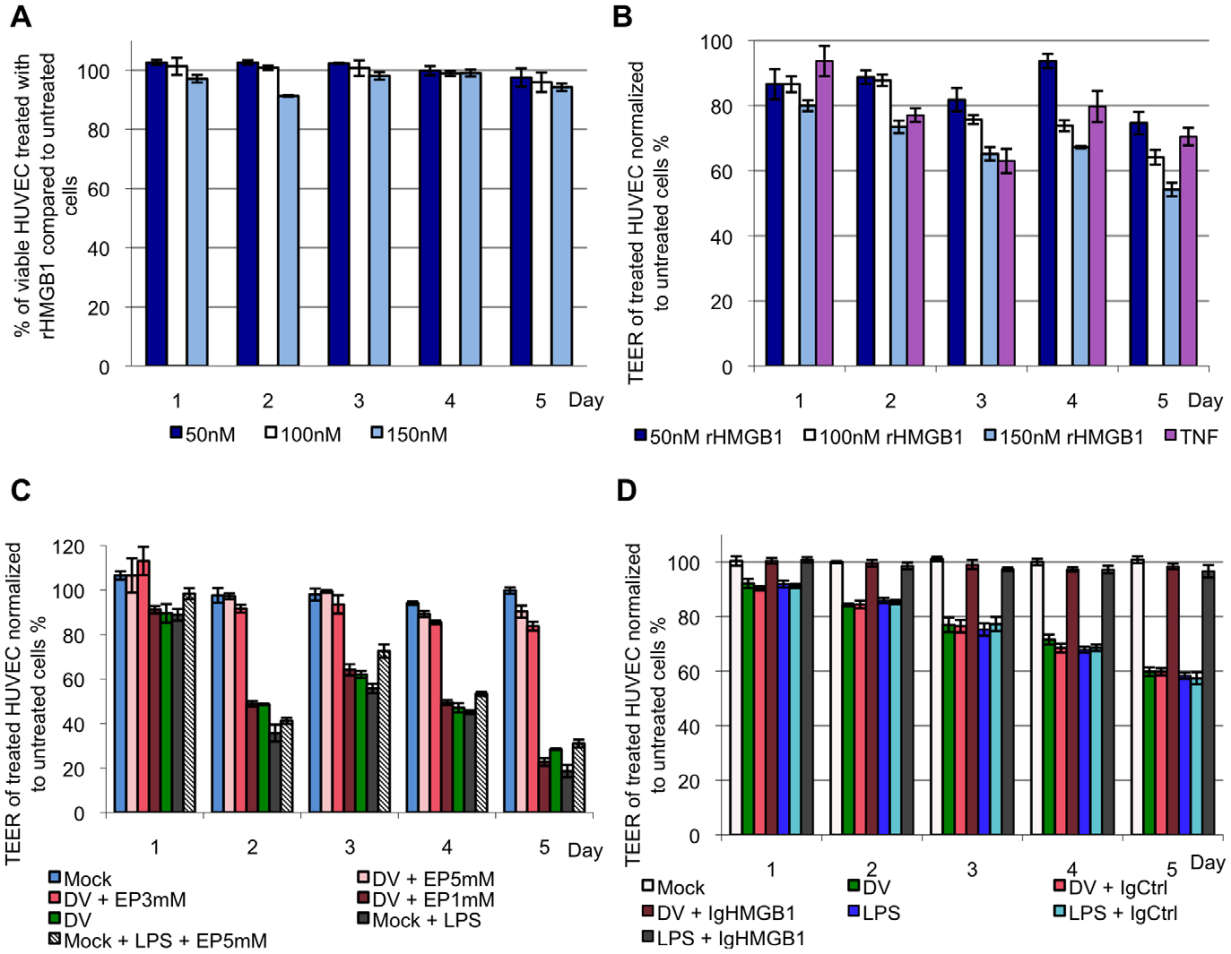
HMGB1 induces vascular leakage in endothelial cells, which can be restored with EP. A, HUVEC was incubated with 50, 100 or 150 nM of rHMGB1 for 5 days and cell viability assay was performed to assess for cytotoxicity induced by rHMGB1 on HUVEC. The percentage of the viable cells from the rHMGB1 treated groups was calculated in relation to the untreated negative control cells. B, 50, 100 and 150 nM of rHMGB1 was added to the confluent HUVEC monolayer to observe for the presence of vascular leakage (indicated by a lowered TEER value) in the endothelial cells. The TEER values of the rHMGB1-treated monolayer were expressed as percentage to the untreated negative control cells. TNF-α, a known inducer of vascular leakage was added to HUVEC monolayer was used as a positive control. C, cell culture supernatant of K562 mock- or DV-infected and EP-treated was collected 3 d.p.i. and added to HUVEC monolayer for 5 days. The TEER of the HUVEC monolayer was measured and the values of the monolayer treated with mock-infected, DV-infected and EP-treated K562 cell culture supernatants were expressed as percentage to HUVEC treated with K562 cell culture medium RPMI-1640 in the absence of any treatment of viral infection. D, mock- or DV-infected or LPS-treated K562 cell culture supernatant was added to HUVEC monolayer for 5 days with chicken anti-HMGB1 neutralizing antibody (IgHMGB1) or chicken HMGB1 control chicken IgY (IgCtrl). The TEER of the supernatant-treated cells was expressed as percentage to HUVEC treated with control RPMI-1640 cell culture medium.

During a natural DV-infection, HMGB1 may be release into the circulatory system, which may have an effect on the vascular integrity, and hence, play a role in the pathogenesis of DHF/DSS. To investigate the role of HMGB1 on vascular permeability in endothelial cells, recombinant human HMGB1 (rHMGB1) was added to HUVEC at a concentration of 50 to 150 nM for 5 days. Cell viability assays was performed to assess the cytotoxicity of rHMGB1 to HUVEC cells and the proportion of viable cells incubated with rHMGB1 was calculated and expressed as a percentage to the negative control cells. HUVEC treated with rHMGB1 were consistently above 90% compared to untreated HUVEC up to day 5 (Fig. 3a). This suggests that rHMGB1 does not induce cytotoxicity in HUVEC at the concentrations tested.

To determine if rHMGB1 affects vascular integrity of endothelial cells, rHMGB1 was added to confluent HUVEC daily for 5 days, and the TEER of HUVEC was recorded each day. A decrease in TEER would indicate an increase in transcellular endothelial permeability. When recombinant HMGB1 was added to HUVEC, a significant reduction in TEER readings were observed as early as 1 day post-treatment when compared to untreated negative control cells (Fig. 3b). The reduction in TEER of HUVEC was consistently across all 5 days of rHMGB1 treatment, with rHMGB1 causing increased vascular leakage in a dose-dependent manner by day 3.

As rHMGB1 was shown to induce vascular leakage in endothelial cells, we postulate that HMGB1 released from DV-infected K562 cells has the same effect on HUVEC. The cell culture supernatant of DV-infected K562 cells was collected 3 d.p.i. and added to HUVEC monolayers daily, followed by measurement of TEER, for up to 5 days. As expected, DV-infected K562 cell culture supernatants induced vascular leakage in the endothelial cells as early as 1 day post treatment (Fig. 3c and d). In addition, TEER readings showed a minimum of 15% reduction from 2 to 5 days of treatment when compared to HUVEC treated with RPMI cell culture medium. Similar trends were also noted in the HUVEC monolayers incubated with non-infected and LPS-treated K562 cell culture supernatants. In contrast, HUVEC incubated with the cell culture media of DV-infected, 5 mM EP-treated K562 cells showed minimal reduction in TEER compared to monolayers treated with mock-infected K562 cell supernatants. This data implies that high concentrations of EP prevent vascular leakage in endothelial cell monolayers through inhibition of HMGB1 release (Fig. 3c). As EP has also been shown to reduce the production of other pro-inflammatory cytokines by endothelial cells [43], which may in turn also affect the TEER, HMGB1 neutralizing antibody was also applied to HUVEC treated with DV-infected K562 cell culture supernatant. Treatment with HMGB1 neutralizing antibody was observedto prevent vascular leakage in HUVEC induced by the cell culture supernatants of DV-infected or LPS-treated K562 (Fig. 3d) whereas treatment with control immunoglobulin Y was unable to inhibit vascular leakage.

### PCAF Mediates HMGB1 Release Upon DV Infection

**Figure 4:**
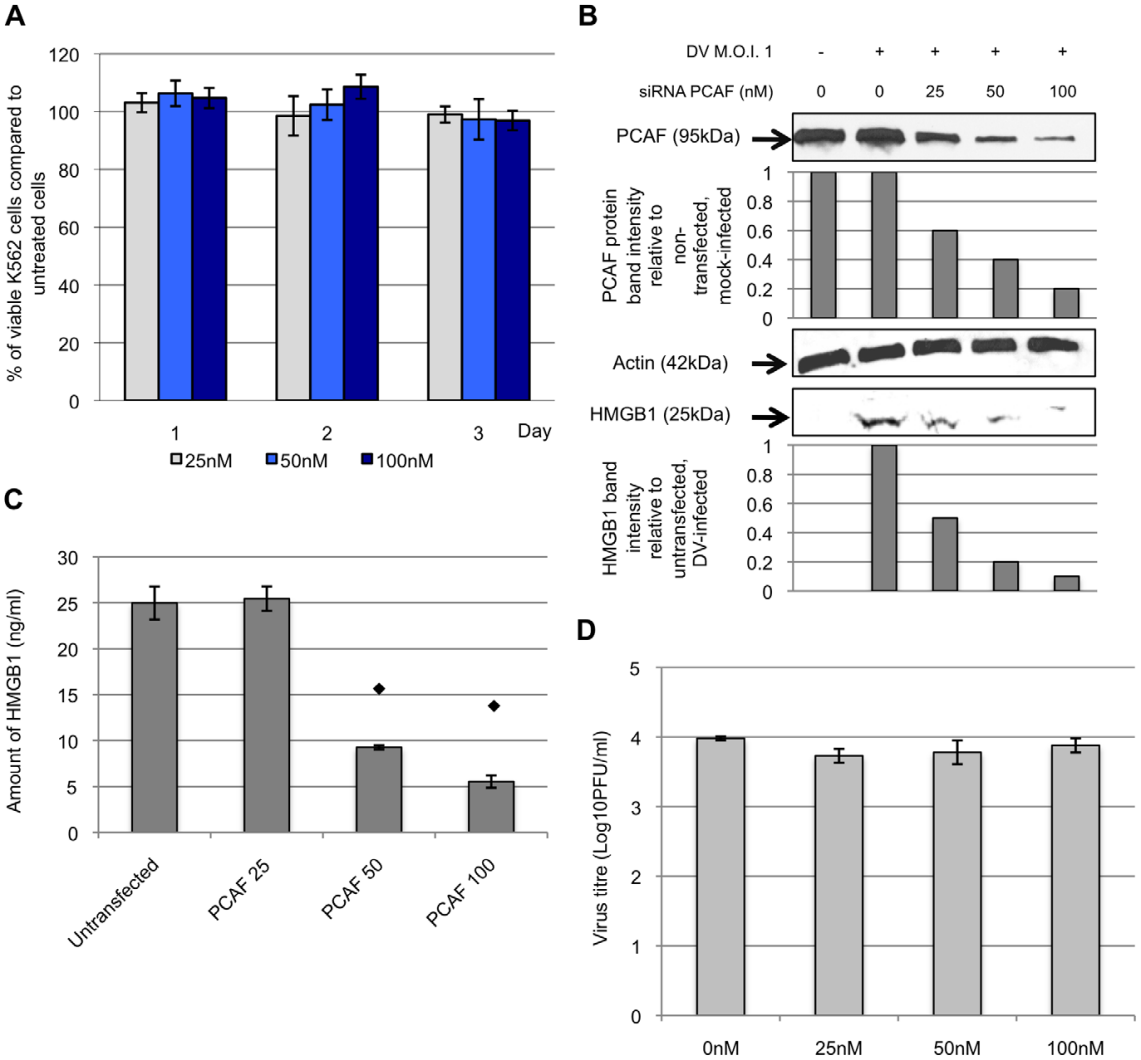
DV mediates HMGB1 release through the involvement of PCAF acetylase complex. A, K562 cells were transfected with 25, 50 or 100 nM of siRNA for 3 days and cell viability assay was performed to evaluation of the cytotoxicity of siRNA transfection on K562 cells. The percentage of the viable transfected cells was expressed as a percentage to that of the non-transfected cells. B, siRNA (concentration of 25 to 100 nM) targeting against PCAF was used to transfect K562 cells. The transfected K562 cells were subjected to DV infection 48 hours post-transfection. The cell lysates and culture supernatants were harvested at 1 d.p.i. and Western blot analysis was then performed to detect for the presence of PCAF and HMGB1, respectively. The PCAF band intensities of the siRNA transfected K562 cells were calculated in relation to the non-transfected mock-infected cells (assigned to a value of 1). Similarly, the band intensities of HMGB1 of the transfected cell culture supernatants were measured in relation to the band intensity of non-transfected DV-infected cell culture media (assigned to value of 1). Actin was used as a control to ensure equal loading. C, ELISA was performed to quantify the amount of HMGB1 released into the supernatants from siRNA transfected (0, 25, 50 or 100 nM of siRNA) and DV-infected K562 cells. ⧫ denotes p-value <0.05 for T-tests comparing the mean amount of HMGB1 detected in siRNA transfected K562 cell culture supernatants to that of the non-siRNA transfected K562 cells. D, K562 cells were transfected with 0, 25, 50 or 100 nM of siRNA for 48 hours before infected with DV. The supernatants were harvested at 1 d.p.i. and plaque assay was performed to quantify the viral yield.

Previous studies have shown that hyperacetylation of HMGB1 in the cell nucleus by PCAF results in the translocation of HMGB1 to the cytosol [29]. To determine if the induction of HMGB1 released upon DV-infection involved PCAF protein, small interfering RNA (siRNA) was utilized to knockdown PCAF in K562 cells. The knockdown of PCAF was assessed by Western blot, with siRNA transfection into K562 cells resulting in a dose-dependent knockdown (Fig. 4b). Furthermore, the concentrations at which PCAF siRNA were used (25 to 100 nM) showed no observable cytotoxicity (Fig. 4a) or inhibition of virus replication (Fig. 4d). In PCAF-knockdown, DV-infected K562 cells, the amount of HMGB1 detected decreased in a dose-dependent manner to the concentration of siRNA used (Fig. 4b and c). At the highest concentration of PCAF siRNA tested (100 nM), the amount of HMGB1 from cells detected was reduced by more than 75%. Taken together, these data suggest that PCAF plays a role in the translocation of HMGB1 from the nucleus during DV infection.

### DV Capsid Protein Induces HMGB1 Release from K562 Cells Involving Acetylation Pathway

**Figure 5:**
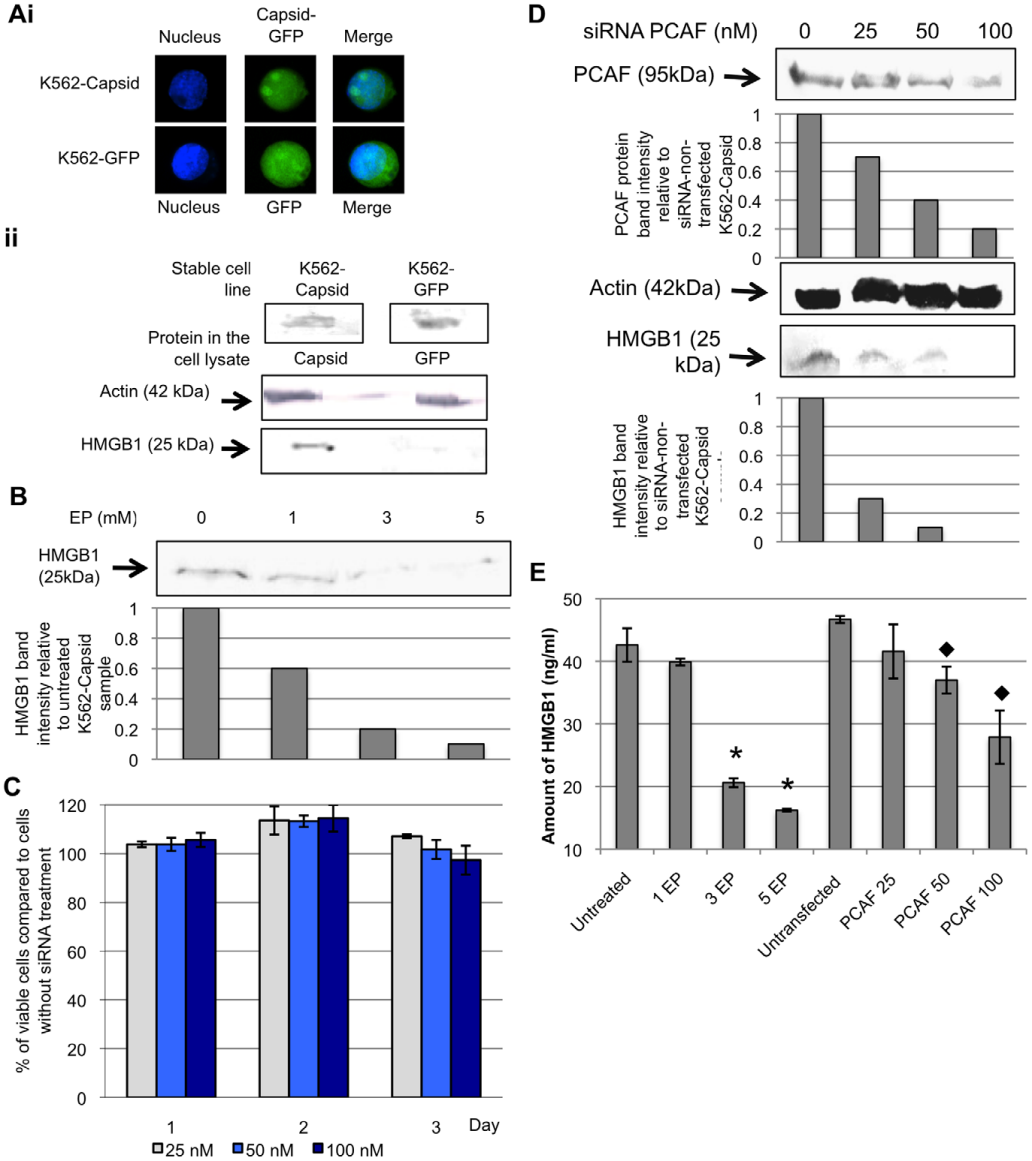
DV capsid protein induces HMGB1 release with the involvement of PCAF acetylase complex. A, K562 cells were transfected with individual plasmid encoding for DV capsid protein or GFP and stable cell line K562-Capsid and K562-GFP were obtained, respectively. IFA and Western blot were performed on the cell lysate to detect for the expression of capsid-GFP fusion protein (39 kDa) and GFP (27 kDa). The cell nuclei were stained blue with DAPI (i) and actin was used as a control to ensure equal loading of the cell lysate (ii). Cell culture supernatants of the stable cell line were harvested and concentrated to probe for the presence of HMGB1 using Western blot. B, stable cell line K562-Capsid was subjected to EP treatment and 3 days post EP treatment, the cell culture were harvested and concentrated to detect for the presence of HMGB1 using Western blot. The band intensities of HMGB1 of the EP treated cell culture supernatants were measured in relation to the band intensity of untreated control cell culture media (assigned to value of 1). C, siRNA was employed to knockdown PCAF protein in the K562-Capsid cells and cell viability assay was performed to evaluate for cytotoxicity upon siRNA transfection. D, K562-Capisd was transfected with siRNA to knockdown PCAF protein and both cell lysate and cell culture supernatants were collected 72 hrs post-transfection. Western blot was performed on the cell lysate and cell culture supernatants to detect for the PCAF and HMGB1, respectively. The band intensities of PCAF protein from the siRNA-transfected cells were compared in relation to the band intensity of untransfected control cell lysate. Actin was used as a control to ensure equal loading of total protein. The band intensities of HMGB1 of siRNA transfected cell culture media was measured in relation to the band intensity of K562-Capsid cells without siRNA. E, the amount of HMGB1 released into the cell culture supernatants from K562-Capsid cells treated with EP or transfected with 0, 25, 50 or 100 nM of siRNA were measured using ELISA. * denotes p-value <0.05 for T-tests comparing the mean amount of HMGB1 detected in EP-treated K562-Capsid cell culture supernatants to that of the untreated K562-Capsid cells. ⧫ denotes p-value <0.05 for T-tests comparing the mean amount of HMGB1 detected in siRNA transfected K562-Capsid cell culture supernatants to that of the non-siRNA transfected K562-Capsid cells.

When K562 cells were treated with UV-irradiated DV (incapable of replication), HMGB1 was observed to translocate from the nucleus to the cytoplasm (Fig. 1a and b) and subsequently gets released into the supernatants as detected by ELISA (Fig. 2d). Furthermore, HMGB1 dominantly reside in the nucleus and DV capsid protein enters the nucleus during infection [44]. This has led to the hypothesis that the DV capsid protein may play a role in mediating HMGB1 release in K562 cells. To determine if DV capsid protein is involved in HMGB1 release from cells, stable cell line was generated in K562 cells expressing DV capsid protein. K562 cells stably transfected with the plasmid vector expressing green fluorescence protein (GFP) was also included in the study. K562-Capsid and K562-GFP stable cell lines were observed to express capsid-GFP fusion protein and GFP, respectively, as shown in Fig. 5ai and ii. Tissue culture fluids from the stable cell-lines K562-Capsid and K562-GFP were harvested and concentrated for the detection of HMGB1 protein. Western blot analyses revealed that K562 cells expressing DV capsid protein release HMGB1 into cell culture supernatants (Fig. 5aii). In contrast, HMGB1 was not detected in the cell culture supernatant of stable cell line expressing GFP.

To further verify the role of DV capsid protein in mediating HMGB1 release, K562-Capsid cells were subjected to EP treatment. Three days post EP treatment, the cell culture supernatants from K562-Capsid cells were harvested and concentrated. An increased concentration of EP treatment directly correlated with a decrease in the amount of HMGB1 detected in cell culture supernatants (Fig. 5b and e). At the highest concentration of EP tested (5 mM), more than 60% reduction in the amount of HMGB1 was detected from K562-Capsid cells.

In addition, experiments were also performed to determine whether DV capsid protein-mediated translocation of HMGB1 from the nucleus is facilitated by PCAF. siRNA was used at a non-cytotoxic concentration (Fig. 5c) to knockdown PCAF protein in K562-Capsid cells, and supernatants were collected 72 hrs post transfection. Western blot and ELISA analyses of HMGB1 clearly show that knockdown of PCAF in K562-Capsid cells results in a dose-dependent decrease in HMGB1 release (Fig. 5d and e). Therefore, DV capsid protein may play a role in mediating HMGB1 release during wild-type DV-infection involving the acetylation pathway.

## Discussion

Besides regulating transcriptional processes [27], HMGB1 also acts as a pro-inflammatory cytokine once released into the extracellular environment during tissue injury and infection [28]. HMGB1 acts by stimulating monocytes to release pro-inflammatory cytokines such as TNF-α, IL-6, and IL-8, all of which have been implicated in the pathogenesis of DHF/DSS [14], [17], [22]. Moreover, HMGB1 has been shown to influence endothelial cell functions such as up-regulation of adhesion molecules, release of cytokines [35] and cytoskeletal rearrangements [45]. In this study, the involvement of HMGB1 was examined during DV-infection of monocytes for that may implicate in the pathogenesis of DHF/DSS.

Studies have shown that epithelial cells infected with a high dose of DV [36] or West Nile virus [46] result in the passive release of HMGB1 from cells into the extraceullular environment. However, we have observed that a low M.O.I. of 0.1 is sufficient to release HMGB1 from cells (data not shown) and this is consistent with observations made in DV-infected DCs [37]. Furthermore, all 4 DV serotypes were capable of inducing the release of HMGB1 from infected K562 cells at a low M.O.I 0.1 (data not shown), indicating that HMGB1 release is a natural consequence of DV infection. These findings were extended to human PBM cells, which were also shown to release HMGB1 in response to DV-infection (Fig. 1).

HMGB1 is released into the extracellular environment as a consequence of DV infection. Contrary to an earlier study conducted by Kamau and colleagues [37], our data showed no inhibition of DV replication in the monocytes in the presence of HMGB1 (Fig. 2d and 4d). The difference in the efficiency of replication may be due to the different DV strains [47] as well as the different cell types used [48]. Treatment with EP was shown to inhibit its release into cell culture media of DV-infected K562 cells (Fig. 2c). This decrease in HMGB1 release by EP was not due to either cell cytotoxicity (Fig. 2a) or inhibition of virus replication (Fig. 2d). Furthermore, 5 mM EP was able to reduce the amount of HGMB1 translocating into the cytoplasm of both DV-infected K562 and PBM (Fig. 2b). These data are consistent with previous observations where EP at a concentration of 5 mM was sufficient to fully inhibit HMGB1 release by LPS-stimulated macrophage cultures [42]. Hence, EP may interfere with the host signalling cascades leading to HMGB1 cytoplasmic localization, prior to its release into the extracellular milieu by DV-infected monocytes.

In response to a pro-inflammatory stimulus, NFκB may impinge on the proteins responsible for HMGB1 release [29] and during DV infection, transcription factor NFκB is activated and translocated to the nucleus [49]. EP may act by reducing NFκB expression and/or attenuating DNA binding activity mediated by the p65 NFκB subunit [50]. This regulates the gene transcription activity of NFκB, leading to the down-regulation of proteins mediating HMGB1 release, thereby inhibiting HMGB1 nuclear-cytoplasmic translocation. DV-infection has also been shown to activate the MAPK pathway [51], which also induces HMGB1 release [29]. EP may also prevent activation of this pathway during DV-infection of K562 cells and PBM by blocking p38 MAPK phosphorylation [42].

HMGB1 released from the monocytes are likely to be in a biological active form [52] as our data (Fig. 3c) are consistent with studies conducted by Wolfson and colleagues [45] where HMGB1 release compromises the vascular integrity of endothelial cells in a dose-dependent manner. In fact, TEER of endothelial cells was reduced with as little as 50–150 nM of rHMGB1 (Fig. 3b). HMGB1 mediates nuclear translocation of NFκB in HUVEC cells via RAGE receptor signalling as the addition of anti-RAGE neutralizing antibodies was observed to prevent vascular leakage in HUVEC in a dosage dependent manner (data not shown). An increase in NFκB-mediated gene transcription could result in the synthesis of pro-inflammatory cytokines, such as TNF-α, which may trigger events leading to enhanced vascular permeability [50]. When applied to HUVEC, the culture supernatants of DV-infected K562 cells resulted in a decrease in TEER of endothelial cells. This may be due to the direct effect of HMGB1 signalling through the RAGE-NFκB pathway as addition of HMGB1 neutralizing antibody was shown to inhibit vascular leakage (Fig. 3d). The disruption of vascular integrity was diminished when endothelial cells were incubated with EP-treated, DV-infected K562 cell culture supernatants (Fig. 3c). EP may work in two ways; (i) inhibiting NFκB and/or p38 MAPK signalling transduction in DV-infected K562 cells thereby preventing the release of HMGB1 (Fig. 2b) as well as other pro-inflammatory cytokines which induces vascular leakage [53], and (ii) controlling DV-induced activation of NFκB and/or MAPK pathways in HUVEC, leading to decreased TNF-α production and HMGB1 release [29].

LPS stimulation of monocytic cells leads to hyper-acetylation of HMGB1 by PCAF, CREB binding protein (CBP) and p300 histone acetylases resulting in its translocation and accumulation in the cytoplasm [29]. siRNA gene silencing of PCAF has resulted in the inhibition of HMGB1 release into the extracellular milieu during DV-infection (Fig. 4b), suggesting that DV induces HMGB1 release through acetylation. While phosphorylation of HMGB1 by calcium/calmodulin-dependent protein kinase (CAMK) IV leads to cytosolic translocation [54], this cannot occur in DV-infected K562 cells, since K562 cells do not express endogenous CAMK IV [55]. Nonetheless, it is possible that DV induces the cytosolic migration of HMGB1 in K562 cells through phosphorylation of HMGB1 by activating classical protein kinase C (cPKC) [56]. The role of other inflammatory signalling mechanisms such as the PI3K, NFκB, and MAPK pathways in mediating the release of HMGB1 is a subject of current debate [56]. Although both K562 and PBM cells share a similar HMGB1 secretory pathway during DV-infection, it is not certain whether acetylation of HMGB1 is the only mechanism contributing to the release of HMGB1 from PBM during DV-infection. Functional studies employing siRNA and specific molecular inhibitors are required to confirm the roles of host proteins in mediating the release of HMGB1 from peripheral blood monocytes during DV-infection.

In this study, both wild type and UV-inactivated DV could induce HMGB1 release in K562 cells. Disruption of the viral (+) ssRNA genome by UV exposure would result in the inhibition of non-structural proteins (NS1, NS2A/B, NS3, NS4A/B, NS5) synthesis, suggesting that DV structural protein(s) play a role in mediating the release of HMGB1. Taken together that HMGB1 predominantly reside in the nucleus and DV capsid protein enters the nucleus during DV-infection [44], we hypothesize DV capsid protein may play a role in mediating the release of HMGB1. Indeed, for the first time to our knowledge, DV capsid protein was found to be the putative viral protein to trigger the release of HMGB1 into K562 cell culture media (Fig. 5a). In addition, the knockdown of PCAF in the nucleus resulted in a diminished release of HMGB1 from DV capsid-expressing K562 cells (Fig. 5d). As such, these data indicate that DV capsid protein may enters the cell nucleus and activate the acetylation pathway of HMGB1, leading to its release into the extracellular environment. Although it is not known if proper uncoating of the UV-irradiated virus could place in the host cells and enters the nucleus, we postulate the capsid protein from the UV-irradiated virus may also enters the nucleus and activate HMGB1 release mechanism, leading to its release into the extracellular environment.

DHF/DSS appears late in dengue illness and HMGB1 may have a role in the progression of DHF/DSS by affecting vascular integrity as well as the secretion of other host cytokines. The release of HMGB1 protein may take place early in the intracellular life cycle of the DV (Fig. 1d) and may also mediate the symptoms of dengue fever. A positive feedback mechanism may enhance the release of HMGB1 [57] as the infection progresses, enabling the protein to reach a level to mediate the symptoms of DHS/DSS.

**Figure 6:**
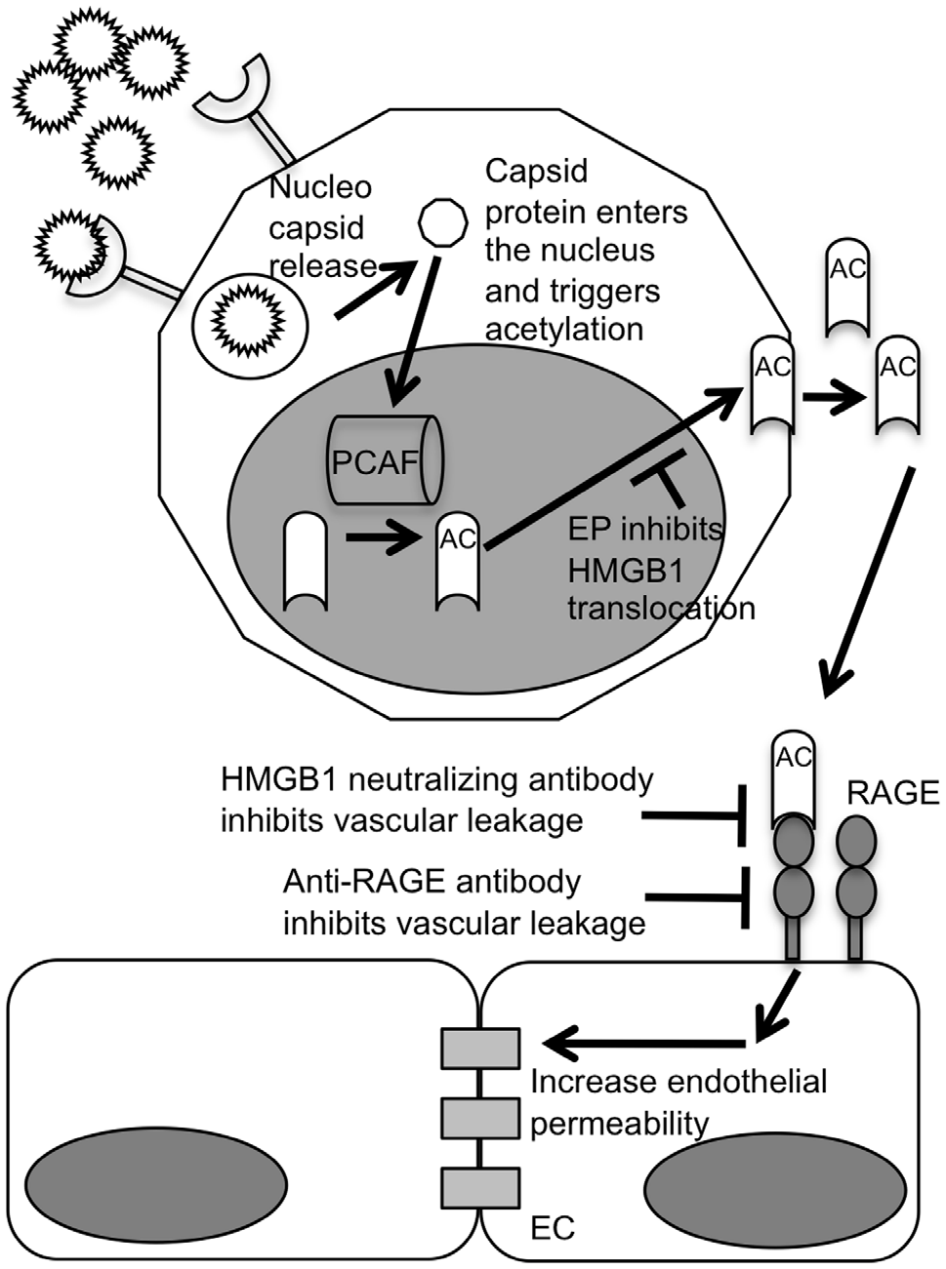
A proposed model of DV capsid protein induces HMGB1 release from monocytes. DV enters the monocytes and uncoat. The capsid protein enters the nucleus and triggers the acetylation of HMGB1 by PCAF complex. HMGB1 then translocates from the nucleus to cytoplasm, a process that can be inhibited by EP. HMGB1 gets released from the monocytes and binds to RAGE receptors on endothelial cells, triggering a signalling pathway which leads to the lost of vascular integrity. The increased in vascular leakage can be repressed by HMGB1-neutraling antibody or anti-RAGE antibody.

From this study, it can be concluded that DV capsid protein triggers the acetylation of HMGB1 after entering the nuclei of monocytes, resulting in the release of HMGB1 into extracellular milieu. HMGB1 is released into the circulatory system, and may act on the endothelium of the blood vessels to induce vascular leakage (Fig. 6), thus causing patients to suffer from DHF/DSS. To confirm the role of DV capsid protein and HMGB1 in the pathogenesis DHF/DSS, further studies are currently investigated suitable animal model.

## Materials and Methods

### Cells and Virus

Human immortalized myelogenous leukemia K562 cells (a kind gift from Emeritus Professor Chan Soh Ha, Department of Microbiology, National University of Singapore) from commercial source, ATCC were grown in RPMI-1640 (Sigma-Aldrich Chemical, St. Louis, MO, USA) supplemented with 10% FCS, while primary human umbilical vein endothelial cells [(HUVEC), Clonetics, Walkersville, USA)] were grown in EGM-2 with 10% FCS and growth supplements provided by the manufacturer (Clonetics, Walkersville, USA). Both cell lines were incubated at 37°C in a 5% CO_2_, humidified incubator. PBM cells were isolated with informed written consent from healthy blood donors as whole blood donation, from the Division of Haematology, Department of Laboratory Medicine, National University Hospital, Singapore and was approved by National University of Singapore-Institutional Review Board (NUS-IRB 10-072E). Following the Ficoll-Paque™ PLUS lymphocyte isolation reagent protocol (GE Healthcare, Piscataway, USA), lymphocytes and monocytes were harvested accordingly. The monocytes were then purified using the MACS monocytes isolation II kit according to the manufacturer’s instructions (Miltenyi Biotec, Bergisch Gladbach, Germany). Cells were cultured in RPMI-1640 medium with 10% heat-inactivated FCS and 1% penicillin/streptomycin (Invitrogen, Carlsbad, California, USA). Dengue virus serotype 2 strain Den2STp7c6 used in this study was propagated in *Aedes albopictus* C6/36 mosquito cells, and plaque assays were carried out to quantify virus titres using BHK-21 cells.

K562 cells were infected with DV at a multiplicity of infection (M.O.I.) of 1 or 10, while PBM cells were infected at a M.O.I. of 1. Ethyl pyruvate [(EP), (Sigma-Aldrich Chemical, St. Louis, MO, USA)] was added to cells at a final concentration ranging from 1 to 5 mM [58]. As a positive control to induce HMGB1 release, LPS (Sigma-Aldrich Chemical, St. Louis, MO, USA) was added to K562 and PBM cells at a concentration of 5 µg/ml [42], [59].

### Nuclear and Cytosolic Extraction

K562 cells (1×10^6^) were infected at M.O.I. of 10, and 3 days post infection (d.p.i.), nuclear–cytoplasmic fractionation was conducted using the NE-PER Nuclear and Cytoplasmic Extraction Reagents kit (Thermo Fisher Scientific, Waltham, MA, USA) according to the manufacturer’s protocol. The extracted fractions were stored at −80°C until further use.

### Indirect Immunofluorescence Microscopy

K562 and PBM cells were infected at M.O.I. of 10 or 1. Three d.p.i., the cells were harvested and fixed using 4% paraformaldehyde followed by permeabilization using 0.01% Triton-X. The cells were double stained with the primary antibodies [1∶1000 dilution for mouse anti-E protein antibody (US biological, Massachusetts, USA) or 1∶500 dilution for rabbit anti-HMGB1 antibody (Abcam, Cambridge, UK)] in a humidified chamber for 1 h at 37°C. Secondary antibodies goat anti-mouse-IgG Alexa Fluor 532 (Invitrogen, Carlsbad, California, USA) or goat anti-rabbit-IgG fluorescein isothiocyanate (FITC)-conjugated (Chemicon, California, USA) at a dilution of 1∶1000 were used. In addition, cell nuclei were stained with 4′,6-diamidino-2-phenylindole (DAPI) at a concentration of 300 nM for 15 mins before washing with PBS. The processed samples were mounted onto glass slides before viewing under the IX-81 epifluorescence microscope (Olympus, Tokyo, Japan) using the Metamorph imaging software program.

### SDS PAGE and Western Blot Analysis

Cell culture supernatants were harvested and concentrated using Amicon® Ultra-4 Centrifugal Filter units (with a cut-off of 10 KDa, Millipore, Schwalbach, Germany) at 3,500 g with the temperature maintained at 4°C. Bradford assay was performed on concentrated supernatants according to the manufacturer’s protocol (Bio-Rad, San Francisco, USA) to determine protein concentrations. The amount of concentrated supernatant for each sample was adjusted to ensure equal loading of total protein before SDS-PAGE separation. The proteins were then transferred onto nitrocellulose membranes using the Trans-Blot SD Semi-Dry Electrophoretic Transfer Cell system (Bio-Rad, San Francisco, USA). To detect HMGB1, rabbit anti-HMGB1 primary antibody was used at a dilution of 1∶500. Alkaline phosphatase-conjugated secondary antibody (Amersham Pharmacia, Buckinghamshire, UK) was used and protein bands were visualized using NBT/BCIP substrate. Band intensity was measured using ImageQuant version 5.2 (Molecular Dynamics, Buckinghamshire, UK). Equivalent proportions of K562 nuclear and cytosolic fractions were loaded onto SDS-PAGE gels for Western blotting to detect for HMGB1, transcription factor (TF)IID (loading control for nuclear fraction) and actin (loading control for cytosolic fraction). Western blotting of cell lysate to detect for PCAF was performed in the similar manner. Mouse anti-TFIID antibody (dilution 1∶500, Santa Cruz Biotechnology, Santa Cruz, CA, USA), mouse anti-actin (dilution 1∶1000, Millipore-Chemicon, Billerica, MA, USA) and mouse anti-PCAF (dilution 1∶100, Santa Cruz Biotechnology, Santa Cruz, CA, USA) were used. For enhanced chemiluminescence detection method, secondary antibody conjugated to horseradish peroxidase at a dilution of 1∶2500 was used together with SuperSignal West Pico Chemiluminescent Substrate (Thermo Fisher Scientific, Waltham, MA, USA).

### Enzyme-Linked Immunosorbent Assay (ELISA)

Cell culture supernatants were collected and the amount of HMGB1 present was quantified using HMGB1 ELISA kit II according to the manufacturer’s protocol (Shino-Test Corporation, Kanagawa, Japan). The sensitivity of this test is 1 ng/ml.

### Cell Viability Assay

Cell viability assays were performed using AlamarBlue reagent (Invitrogen, Carlsbad, California, USA) according to the manufacturer’s instruction. Living cells maintain a reducing environment in the cytosol and convert resazurin in the AlamarBlue reagent to resorufin. One-tenth volume of the AlamarBlue reagent was added to cells and incubated for 3 hours at 37°C. Fluorescence reading of resorufin was measured at excitation wavelength of 570 nm, and emission wavelength at 585 nm. The fluorescence readout of treated cells was expressed as a percentage to that of untreated cells.

### Transendothelial Electrical Resistance

HUVEC were seeded on transwell inserts (3.0 µm pore, 6.5 mm diameter, Millipore, Schwalbach, Germany) for 2 days to allow monolayers to reach confluency. Recombinant HMGB1 (rHMGB1) was added to the upper chamber of the transwell inserts daily, and the transendothelial electrical resistance (TEER) of the HUVEC monolayers were measured using Millicell-ERS volthohmeter (Millipore, Schwalbach, Germany). Cell culture supernatants of DV-infected K562 cells treated with or without EP were collected at 3 d.p.i. and concentrated using Amicon® Ultra-4 Centrifugal Filter units (with a cut-off of 10 KDa, Millipore, Schwalbach, Germany). RPMI-1640 cell culture medium was concentrated in a similar manner and used as a control supernantant. Bradford assay (Bio-Rad, San Francisco, USA) was performed to determine the total protein concentration in each concentrated culture supernatant before adding the same amount of protein to confluent HUVEC. Chicken anti-HMGB1 neutralizing antibody or chicken HMGB1 control chicken IgY (IBL International, Hamburg, Germany) was added to the confluent monolayer to achieve a final concentration of 100 µg/ml. TheTEER of the monolayers was measured daily.

### Transfection of Cells

K562 cells (1×10^6^) were transfected with Dharmacon siGENOME SMARTpool siRNA targeting P300/CBP-associated factor (PCAF) (L-005-55) (Lafayette, Colorado, USA) at a concentration range of 25 to 100 nM using DharmaFECT 1 (Dharmacon, Lafayette, Colorado, USA). Twenty-four hours post transfection, cells were washed with PBS and incubated for another 48 hrs to ensure efficient knockdown of PCAF.

The dengue capsid gene was cloned into the mammalian expression vector pcDNA 6.2/C-EmGFP (Invitrogen, Carlsbad, California, USA) in frame with a GFP tag. K562 cells were seeded at a density of 2.5×10^5^ cells/ml on 6-well plates (Nunc, InterMed, Denmark) overnight. The plasmids pCapsid and pGFP (as a control vector) were individually transfected into K562 cells using Lipofectamine LTX reagent (Invitrogen, Carlsbad, California, USA) according to the manufacturer’s protocol. In brief, 15 µg of DNA were added to 3 ml of Opti-MEM (Invitrogen, Carlsbad, California, USA) mixed with 15 µl of PLUS reagent and 60 µl of Lipofectamine LTX reagent. Ten µg/ml blasticidin (Invitrogen, Carlsbad, California, USA) were added to the wells 48 hrs post transfection to selected for transfected cells and to generate the stable cell lines (K562-Capsid and K562-GFP). The cell culture supernatants from the stable clones were collected for the detection of HMGB1 by Western blot.
